# Fuzzy Optimization for the Remediation of Ammonia: A Case Study Based on Electrochemical Oxidation

**DOI:** 10.3390/ijerph18062986

**Published:** 2021-03-14

**Authors:** Angelo Earvin Sy Choi, Benny Marie B. Ensano, Jurng-Jae Yee

**Affiliations:** 1 Chemical Engineering Department, De La Salle University, 2401 Taft Avenue, Manila 0922, Philippines; 2 University Core Research Center for Disaster-free and Safe Ocean City Construction, Dong-A University, Busan 49315, Korea; bmensano@dau.ac.kr; 3 Department of Architectural Engineering, Dong-A University, Busan 49315, Korea

**Keywords:** ammonia, electrochemical oxidation, fuzzy optimization, energy consumption, operational cost

## Abstract

This case study covers the application of the fuzzy optimization in simultaneously satisfying various constraints that include the compliance of ammonia and nitrate concentrations with stringent environmental standards. Essential components in the multi-criteria decision-making analysis is in the utilization of the Box-Behnken design (BBD) response equations, cost equations and the cumulative uncertainty of response towards the sodium chloride dosage, current density and electrolysis time parameters. The energy consumption in the electrochemical oxidation of ammonia plays an essential role in influencing the total operating cost analysis. The determination of boundary limits based on the global optimum resulted in the complete ammonia removal and USD 64.0 operating cost as its maximum boundary limits and the 40.6% ammonia removal and USD 17.1 as its minimum boundary limits. Based on the fuzzy optimal results, the overall satisfaction level incurred a decrease in adhering with a lower ammonia standard concentration (10 mg/L at 80.3% vs. 1.9 mg/L at 76.1%) due to a higher energy consumption requirement. Global optimal fuzzy results showed to be highly cost efficient (232.5% lower) as compared to using BBD alone. This demonstrates the practicality of fuzzy optimization applications in the electrochemical reactions.

## 1. Introduction

Excessive discharge of nutrients into water bodies, mainly due to anthropogenic activities [1], has profound global environmental and health effects. High levels of nitrogen contributes to algal bloom in receiving surface waters resulting in depletion of dissolved oxygen and production of toxins that are harmful to aquatic organisms [2]. Meanwhile, ammonia (NH_3_ and/or NH_4_^+^) and nitrates (NO_3_^-^) in drinking water sources may cause blue baby syndrome, childhood diabetes, acute respiratory tract infections, and cancer when ingested in excess [3]. As a consequence, the European Water Framework Directive (2000/60/EC) set a wastewater discharge limit of 10–15 mg N L^−1^ in ecologically sensitive areas [4]. In 2013, the US Environmental Protection Agency (USEPA) adjusted the freshwater quality criteria for ammonia based on the toxicity to mussels and the non-pulmonate snails, such that, at pH 7.0 and 20 °C, the criterion magnitudes are 17 and 1.9 mg total ammonia nitrogen (TAN) L^−1^ for a 1-h (acute) and 30-day (chronic) average duration, respectively [5]. Moreover, a maximum permissible limit of 10 and 11.3 mg NO_3_-N L^−1^ in public drinking water supplies were established by USEPA and the World Health Organization (WHO), respectively [3].

Biological process, ion exchange, chemical precipitation, and air stripping are among the ammonia removal technologies that have been extensively applied in municipal and industrial wastewaters [6]. Recently, particular interest has been given to the electrochemical oxidation of ammonia due to its promising potential in alleviating energy and environmental problems (i.e., sensors [7], fuel cells [8], and wastewater treatment applications [9]). This method boasts several advantages over other water remediation techniques, which include enhanced efficacy, minimal sludge generation, and small footprint [10]. As a result, it has been employed in different types of wastewater namely landfill leachate [11], tannery wastewater [12], and lead smelting wastewater [13].

Electro-oxidation of ammonia occurs via direct and/or indirect oxidation. During direct oxidation, chemisorbed ammonia is decomposed into harmless nitrogen gas by direct anodic electron transfer. In the indirect oxidation, chloride ion (Cl^−^) is added for in situ generation of active chlorine species that mediate the conversion of ammonia and other reductive by-products into nitrogen [14]. Previous studies have demonstrated that chloride ion concentration, electrolysis time, and current density are among the critical parameters that influence the efficiency and rate of ammonia electro-oxidation. High chloride ion concentration and prolonged electrolysis time can generate more active chlorine species to mediate the ammonia oxidation, while an increase in current density improves the electron transfer rate on the electrode surface thereby accelerating the ammonia oxidation process [13]. However, possible side reactions (i.e., oxygen evolution and toxic nitrate formation) and the reduction of current efficiency may occur at elevated current density and extended electrolysis time [15]. In addition, carcinogenic chloramines may also be produced depending on the chloride ion concentration [16]. Therefore, the optimization and control of these process parameters are imperative to minimize such occurrences.

Li et al. [10] studied the application of response surface methodology (RSM), a multivariate statistical and mathematical tool, on the electrochemical oxidation of ammonia in an undivided cell using Ti/IrO_2_ anode and Cu-Zn cathode. Box-Behnken design (BBD) was utilized to determine the optimum conditions that would simultaneously yield the highest ammonia removal and lowest nitrate production. The optimum values obtained were 0.31 g L^−1^, 42.75 mA cm^−2^, and 100.64 min for NaCl dosage, current density, and electrolysis time, respectively, resulting in a complete removal of ammonia and final nitrate concentration of 1.1 mg L^−1^. Using RSM, Li et al. [10] has also successfully identified the effects of experimental parameters and their potential interactions on the removal of ammonia and nitrate via electrochemical oxidation. However, it failed to incorporate the cumulative error of uncertainty and the operating cost of the process. In practical methods, the measurement of the uncertainty is essential due to the incorporation of inevitable errors associated with each of the equipment used in the experimental runs [17,18]. Specifically, the cumulative uncertainty of the results in the system that contributes to the electro-oxidation of ammonia by the variables of NaCl dosage, current density, and electrolysis time needs to be further assessed. In addition, a cost analysis should be considered since the process of ammonia and nitrate removal via electrochemical oxidation consumes electricity and chemicals. This is essential in order to evaluate the feasibility of the process for practical and environmental applications.

Fuzzy optimization is an artificial intelligence-based modeling technique widely used for process optimization and control [19]. It simulates human reasoning and decision-making using truth degrees as a mathematical basis to produce a reliable and definite output from an incomplete, vague or imprecise input [20]. This gives fuzzy logic an edge over the traditional computer logic that cannot manipulate data representing subjective opinions. Meanwhile, both stochastic and fuzzy programming approaches can be employed for optimization techniques under uncertainty [21]. However, stochastic modeling requires an enormous data processing, i.e., vague data are condensed into average data, to create a model that adequately maps the real problem [22]. In fuzzy optimization modeling, no extensive information processing is needed since the vague data are precisely modeled by fuzzy numbers and fuzzy intervals using only the available information. Subsequently, the decision maker will decide based on the fuzzy optimal solution which objective oriented additional information has to be considered [22]. This stepwise improvement of data representation and solution makes fuzzy optimization more practical than stochastic optimization. Hence, fuzzy optimization has been utilized in various water treatment applications such as biological treatment [23], anaerobic co-digestion [24], electrocoagulation [25], oxidative desulfurization [26], and superabsorbent synthesis [27]. However, based on extensive literature review, no study has been reported yet on the application of fuzzy optimization in the electrochemical oxidation of ammonia.

Fuzzy optimization can be used to derive a global optimal solution from a multi-objective decision-making problem unlike RSM that considers only local optimization and only has a uni-model objective function [28,29]. There are multiple local optimal points that can be generated in RSM but the fuzzy optimization through the Lingo 18.0 software has a global solver that determines the global optimum. Therefore, multi-objective optimization utilizing the concept of fuzzy logic is an attractive tool to determine the most favorable compromise solution (best ammonia removal at the least possible operating cost). Hence, this study modeled the ammonia electro-oxidation by the incorporation of the optimization and cost analysis using RSM as part of the objective function. The cumulative uncertainty of the results was added for the optimization process to make certain that the errors associated to the variables are accounted for. Moreover, the material usage and energy consumption of the factors affecting the electrochemical oxidation process were determined as the basis for the resulting total operating cost.

## 2. Materials and Methods

### 2.1. Experimental Method

**Figure 1:**
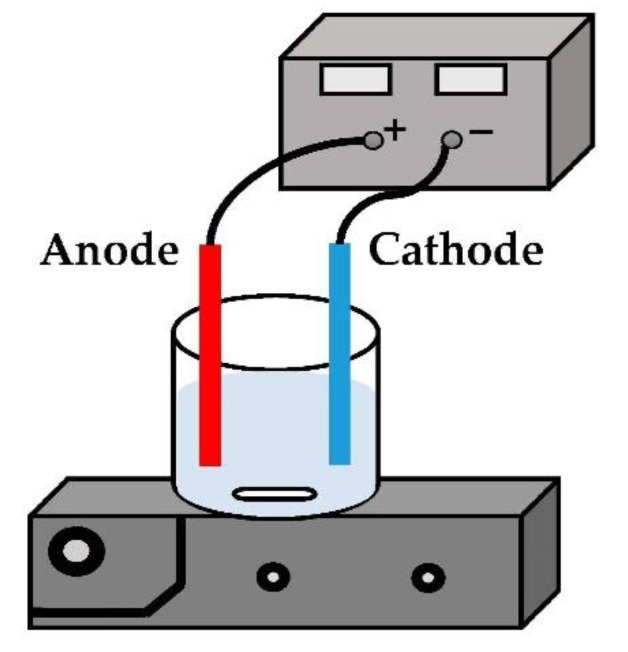
Schematic diagram of the electrochemical oxidation system.

Based on the study of Li et al. [10], ammonia electrochemical oxidation was conducted using a cylindrical undivided electrolytic cell (400 mL working volume) with Ti/IrO_2_ plate and Cu-Zn as the anode and cathode, respectively. The dimensions of the electrodes were the same (15 × 5 cm) with immersed areas of 40 cm^2^. Approximately 300 mL of a synthetic ammonia solution that comprised of ammonia-N ((NH_4_)_2_SO_4_, 100 mg L^−1^), sodium sulfate (Na_2_SO_4_, 0.50 g L^−1^), and sodium chloride (NaCl, 0–1.0 g L^−1^) was poured into the electrochemical cell. The electrodes were placed inside the cell at a maintained electrode gap of 8 mm. They were then connected to a DC power supply that has a voltage range of 0–240 V and a current range of 0–25 A. The specified current density was applied and the electrolysis proceeded under room temperature (25 ± 2 °C). Lastly, 1.5 mL samples were taken from the electrochemical cell at pre-determined time intervals for the analysis of ammonia and nitrate concentrations. A schematic diagram of the experimental setup is illustrated in Figure 1.

### 2.2. Fuzzy Multi-Objective Optimization Method

**Figure 2:**
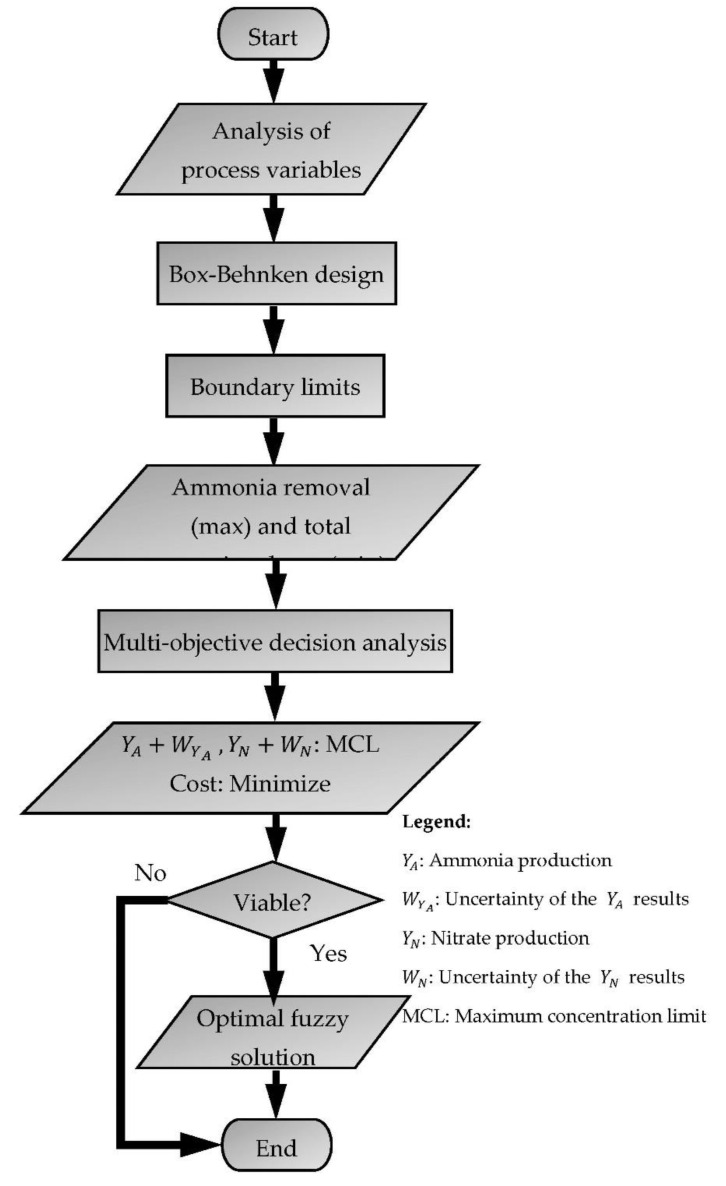
Multi-objective fuzzy optimization process flowchart.

A flowchart for the algorithm of the fuzzy optimization process used in this study is presented in Figure 2. The resulting BBD was derived from the study of Li et al. [10] as the basis for the case study in this research paper. The determination of boundary limits followed by the multi-objective fuzzy optimization were applied to identify the best possible parametric conditions that ascertain maximum ammonia oxidation at the lowest total operating cost. The viability of the results was based on the convergence of the equation towards satisfying the maximum contamination limits of ammonia and nitrate. A viable result would lead to an optimal fuzzy solution (highest ammonia oxidation at the least cost) generated from the fuzzy optimization algorithm. The process parameters (including the scale of the experiment) involved in the electro-oxidation of ammonia, including the corresponding cost factors, are listed in Table 1. Table 2 summarizes the data derived from the study of Li et al. [10] with the inclusion of the cumulative uncertainty of results. A linguistic value representing the degrees from completely false (0) to completely true (1) was utilized to interpret the reasoning approach in the fuzzy optimization analysis [30]. Past studies claimed that the fuzzy constraints of non-linear models might be difficult to run in the mathematical software such as CPLEX and Lingo [31,32]. However, for the Lingo software, this is dependent on the type of license subscription. In this study, the optimization was performed using Lingo 18.0 (Lindo Systems, Chicago, IL, USA), an advanced modeling software that uses a global optimizer for non-linear programming. The educational license for the utilized Lingo program includes global and non-linear solvers with no limitation in its number of variable usages. This made Lingo 18.0 a powerful solver to obtain global optimal solutions for a non-linear program model class. The typical elapsed runtime to achieve the global optimal solution in a non-linear model is around 0.50 s.

#### 2.2.1. Determination of Boundary Limits 

In this study, the boundary limits were determined using model equations of ammonia removal and its operating cost based on material usage and energy consumption. The identified boundary limits were then utilized in the multi-objective fuzzy optimization. 

The first objective function to identify the boundary limits is designated by the maximization of the ammonia removal (*Y*, %) together with its cumulative uncertainty $\left(W_{Y_{A}}\right)$ in Equation (1). This enabled the determination of the upper boundary limit of ammonia removal and operating cost. On the other hand, another objective function for the boundary limit identification is to minimize the operating cost (*C_T_*: USD) that accounts for the material usage and energy consumption in the electrochemical removal process of ammonia, as shown in Equation (2). This in turn designated the lower boundary limits for the removal of ammonia and its associated total cost.

(1) $$Y+W_{Y_{A}}$$

(2) $$\min C_{T}$$

Equations (3)–(8) are constraints that must be subjected to the objective functions for an appropriate identification of boundary limits. The percentage removal of ammonia (*Y*: %) is given in Equation (3), while the response equations of the production of ammonia (*Y_A_*: mg/L) and nitrate (*Y_N_*: mg/L) are formulated in Equations (4) and (5), respectively. Meanwhile, Equations (6) and (7) account for the cumulative uncertainty analysis of ammonia removal $\left(W_{Y_{A}}:\;\mathrm{mg}/L\right)$ and nitrate production $\left(W_{Y_{N}}:\;\mathrm{mg}/L\right)$, respectively. Equation (8) is the constraint for the objective function that considers the total operating cost in terms of the material usage and electric cost. On the other hand, Equation (9) shows the calculation for the energy consumption (*E_C_*: kWh/kg-NH_3_) which is essential for computing the electrical cost towards the electrochemical oxidation system in ammonia removal. The energy consumption is based on the voltage (*V*: V), current (*I*, A), reaction time (∆t: h), working volume (v: m^3^), and ammonia removal ($∆NH_{3}$: mg/L). Lastly, Equation (10) appropriately allocates the feasible regions of parameters towards the response.

(3) $$Y=\frac{100-Y_{A}}{100}$$

(4) $$Y_{A}=\alpha_{0}+\overset{3}{\underset{i=1}{\sum }}\alpha_{i}X_{i}+\overset{3}{\underset{i=1}{\sum }}\overset{3}{\underset{j=i+1}{\sum }}\alpha_{ij}X_{i}X_{j}+\overset{3}{\underset{i=1}{\sum }}\alpha_{ii}X_{i}^{2}$$

(5) $$Y_{N}=\beta_{0}+\overset{3}{\underset{i=1}{\sum }}\beta_{i}X_{i}+\overset{3}{\underset{i=1}{\sum }}\overset{3}{\underset{j=i+1}{\sum }}\beta_{ij}X_{i}X_{j}+\overset{3}{\underset{i=1}{\sum }}\beta_{ii}X_{i}^{2}$$

(6) $$W_{Y_{A}}={\left(\overset{3}{\underset{i=1}{\sum }}\frac{\partial Y_{A}}{\partial X_{i}}W_{X_{i}}\right)}^{\frac{1}{2}}$$

(7) $$W_{Y_{N}}={\left(\overset{3}{\underset{i=1}{\sum }}\frac{\partial Y_{N}}{\partial X_{i}}W_{X_{i}}\right)}^{\frac{1}{2}}$$

(8) $$C_{T}=\sum C$$

(9) $$E_{C}=\frac{V\cdot I\cdot ∆t}{1000\cdot v\cdot ∆NH_{3}}$$

(10) $$\overset{3}{\underset{i=1}{\sum }}X_{i}^{L}\leq \overset{3}{\underset{i=1}{\sum }}X_{i}\leq \overset{3}{\underset{i=1}{\sum }}X_{i}^{U}$$

#### 2.2.2. Multi-Objective Decision-Making through Fuzzy Optimization 

A multi-criteria decision analysis is essential to evaluate the optimal solutions and come up with the best feasible solution [33]. To solve a multi-objective decision-making problem, a fuzzy mathematical programming approach was applied [28,29]. The objective function is to maximize the overall level of satisfaction ($\lambda_{O}$: %) given in Equation (11) using the concept of max-min aggregation, while simultaneously optimizing the degree of satisfaction. That is, the degree of satisfaction for the ammonia removal and total operating cost must satisfy the overall satisfaction.

(11) $$\lambda_{O}\leq \lambda_{k}$$

Equations (12)–(15) refer to the fuzzy constraints utilized to obtain the best compromise solution in the multi-objective criteria. Specifically, Equations (12) and (13) are designated as the linear membership function for maximizing the removal of ammonia ($\lambda_{1}$: %) and minimizing the total operating cost ($\lambda_{2}$: %), respectively. Equation (14) specifies the limiting constraint in relation to the standard regulatory limits of ammonia and nitrate contaminants. Finally, Equation (15) indicates the feasible region of the degree of satisfaction for the fuzzy optimization analysis. The designated fuzzy parameters include the upper and lower bounds of the ammonia removal as ${\left(Y+W_{Y_{A}}\right)}^{U}$ and ${\left(Y+W_{Y_{A}}\right)}^{L}$, respectively, and the upper and lower bounds of the operating cost as $C_{T}^{U}$ and $C_{T}^{L}$, respectively. Additionally, the fuzzy optimization process is also subjected to the constraints in Equations (3)–(10).

(12) $$\lambda_{1}=\frac{\left(Y+W_{Y_{A}}\right)-{\left(Y+W_{Y}\right)}^{L}}{{\left(Y+W_{Y_{A}}\right)}^{U}-{\left(Y+W_{Y_{A}}\right)}^{L}}$$

(13) $$\lambda_{2}=\frac{C_{T}^{U}-C_{T}}{C_{T}^{U}-C_{T}^{L}}$$

(14) $$Y+W_{Y_{A}}\;\mathrm{and}\;Y_{N}+W_{Y_{N}}\leq \gamma$$

(15) $$\lambda^{L}\leq \lambda_{O}\leq \lambda^{U}$$

## 3. Results and Discussion

### 3.1. Analysis of NaCl Dosage towards Ammonia Removal and Its Operating Cost 

The parametric factor of NaCl dosage is essential in investigating ammonia removal via the electrochemical process as this directly relates to the concentration of active chlorine species. During the indirect oxidation of ammonia, chlorine gas (Cl_2_) is generated at the anode in the presence of chloride ions [34]. This chlorine gas easily hydrolyzes in the solution to form HClO which in turn is converted to ClO^–^ [10]. Both HClO and ClO^–^ are active chlorine species capable of oxidizing ammonia to nitrogen gas or nitrate. Consequently, the nitrate ions are reduced at the cathode yielding nitrite, nitrogen or ammonia [10]. Due to the presence of the active chlorine species in the solution, the nitrite and ammonia are quickly oxidized back until all the ammonia and by-products are converted to nitrogen [34].

**Figure 3:**
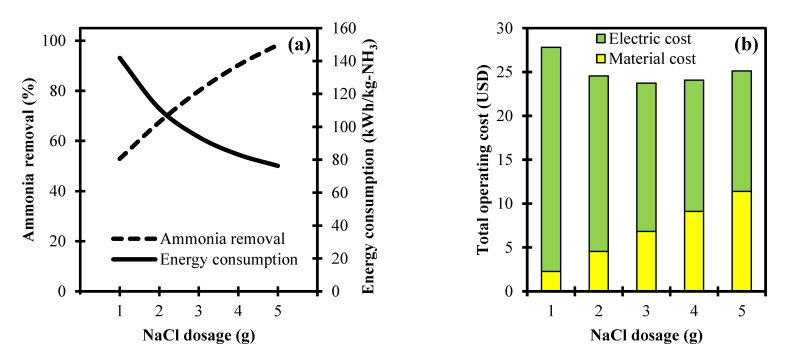
Process variable analyses of NaCl dosage towards: (**a**) Ammonia removal, energy consumption, and (**b**) total operating cost in an electrochemical system.

Figure 3a illustrates the interdependency of ammonia removal and the energy consumption at different NaCl dosage and constant parametric settings of 10 mA/cm^2^ and 75 min for current density and electrolysis time, respectively. A low NaCl dosage of 1 g results in a 52.9% ammonia removal. This is due to the lack of chloride concentration that can generate ClO^–^. However, it is observed that an increasing trend of the NaCl dosage from 1 to 5 g is able to effectively remove ammonia in the electrochemical oxidation process from 52.9% to 98.3%. This is associated with higher concentrations of ClO^–^ that contain a high oxidative capability which influences the oxidation of ammonia [10]. In the aspect of energy consumption, the NaCl dosage of 1 to 5 g shows a declining trend in the energy consumption from 141.9 to 76.3 kWh/kg-NH_3_. This gives the implication that ammonia removal is more efficient at a higher NaCl dosage due to a simultaneous attainment of a higher removal rate at a lower consumed amount of energy.

The analysis of the total operating cost that considers the material cost and electric cost associated with the NaCl dosage and the energy consumption, respectively, is depicted in Figure 3b. Based on the results, an incremental trend of NaCl dosage from 1 to 5 g increases the material cost from USD 2.3 to 11.4. This is due to the utilization of a higher amount of NaCl in the electrochemical oxidation of ammonia. In contrast, the electric cost declined from USD 25.5 to 13.7 for the NaCl dosage of 1 to 5 g, respectively. This can be attributed to the decrease in energy consumption as the NaCl dosage increased, as consistently presented in Figure 3a. In the summative analysis of the total operating cost, a decremental trend (USD 27.8 to 23.7) is observed from 1 to 3 g of the NaCl dosage in accounting both the material and electric costs. This is attributed to a substantial decrease in the energy consumption needed for ammonia removal at a higher NaCl dosage. However, increasing the dosage further from 3 to 5 g yields an increasing trend in the total operating cost from USD 23.7 to 25.1. This is due to the influence of a high material cost from the use of NaCl in this range.

### 3.2. Analysis of Current Density towards Ammonia Removal and Its Operating Cost 

**Figure 4:**
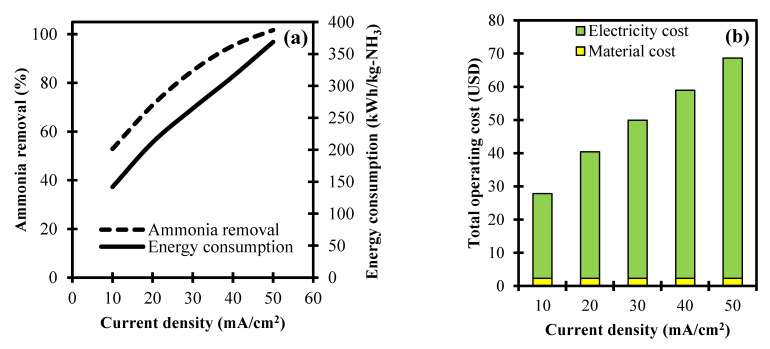
Process variable analyses of current density towards: (**a**) Ammonia removal, energy consumption, and (**b**) total operating cost in an electrochemical system.

The current density is another vital parameter in the electrochemical oxidation of ammonia. This variable controls the electron losing rate in the chloride ions which consequently affects the ammonia oxidation rate [34]. Figure 4a shows the dependent variable of current density and the independent variables of ammonia removal, as well as the total operating cost at a constant NaCl dosage of 1 g and electrolysis time of 75 min. 

Results indicate that ammonia removal is limited to 52.9% in a current density setting of 10 Ma/cm^2^. This is due to the slow oxidation reaction rate at a low-level current density [16]. Conversely, increasing the current density to 50 Ma/cm^2^ shows an upward trend leading to the complete removal of ammonia. This is attributed to a higher tendency of the hydroxyl ions to be attracted towards the anode at a more intensive current density [10]. The current density in higher levels also increases the evolution of oxygen and formation of ClO^–^ that enhances the ammonia removal in the electrochemical reaction system [16]. Furthermore, the required energy consumption is observed to increase from 141.9 to 368.8 kWh/kg-NH_3_ along with the range of the current density from 10 to 50 Ma/cm^2^, respectively. The attained energy consumption is attributed to the minimum energy requirement for each of the current density levels needed to overcome the resistance over the Nernst potential and concentration polarization in the electrochemical reactions [35].

Figure 4b represents the total operating cost for the electrochemical oxidation of ammonia at varying current densities. For the material cost, this is constant at USD 2.3 at different levels of the current density due to setting the NaCl dosage at a constant value of 1 g. On the other hand, the electric cost increased from USD 25.5 to 66.4 at the current density levels from 10 to 50 Ma/cm^2^, respectively. This is due to the higher energy consumption needed to effectively remove ammonia at a higher current density, as consistently illustrated in Figure 4a. In a cumulative evaluation of the material and electric costs, the total operating cost leads to an upward trend from USD 27.8 to 68.7. Based on the results, the total operating cost is highly dependent on the current density due to the strong influence of the energy consumption requirement for ammonia removal. 

### 3.3. Analysis of Electrolysis Time towards Ammonia Removal and Its Operating Cost 

**Figure 5:**
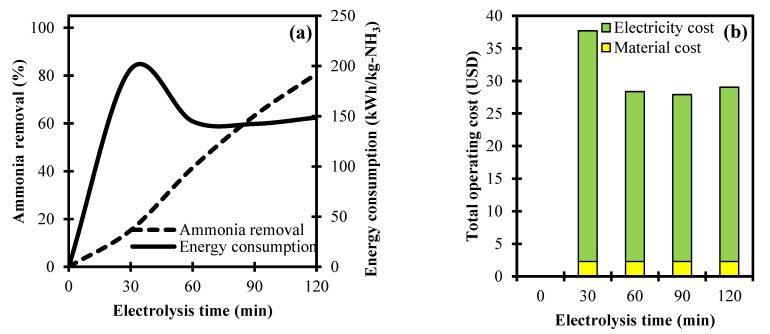
Process variable analyses of electrolysis time towards: (**a**) Ammonia removal, energy consumption, and (**b**) total operating cost in an electrochemical system.

An important parameter variable to consider in the electrochemical oxidation of ammonia is the time of electrolysis. Specifically, the electrolysis time in the electrochemical system is directly associated with the charge loading that stimulates oxidation reactions [36]. Figure 5a shows the distinct effect of electrolysis time at constant parameters of 1 g NaCl dosage and 10 Ma/cm^2^ current density against ammonia removal and energy consumption.

A limited electrolysis time corresponds to a low removal rate due to the inadequate reaction time for the complete oxidation of ammonia in the system. It is observed that prolonging the electrolysis time from 0 to 120 min resulted in an increasing trend of ammonia removal from 0% to 80.7%, respectively. This is attributed to the enhanced generation of ClO^–^ that aids in the removal of ammonia at a longer electrolysis time [37]. Based on the energy consumption, a marked increase of 196.7 kWh/kg-NH_3_ is observed at 30 min (electrolysis time) but only achieved a 15.3% ammonia removal. In relation to Equation (9), the principle of a high consumed energy refers to the low removal rate of the ammonia contaminant that proves to be inefficient at a short electrolysis time. At the electrolysis time of 60 to 120 min, the energy consumption is in the range of 142.3 to 148.6 kWh/kg-NH_3_. The small difference in energy consumption implies that it achieved its equilibrium in this interval. Thus, the best condition in the given set of range for the electrolysis time is at 120 min due to having the highest ammonia removal rate at a relatively low energy requirement. 

The results for the electrolysis time against the total operating cost (material and electric cost) is depicted in Figure 5b. The material cost for the given set of parameters is fixed at USD 2.3. This is in relation to setting the NaCl dosage constant at 1 g throughout the testing range of the time of electrolysis. In line of the electricity cost, an electrolysis time of 30 min attained its highest cost of USD 35.4. This is due to a large amount of energy consumption needed at 30 min, as consistently shown in Figure 5a. At the electrolysis times of 60, 90, and 120 min, the obtained electric costs are USD 26.1, 25.6, and 26.8, respectively. The close values of these costs are associated to a small change in the energy consumption from 60 to 120 min. Additionally, the total operating cost is observed to have a strong influence towards the energy requirement due to its consideration of the simultaneous effects of electrolysis time and the removal rate of ammonia in the electrochemical oxidation process. 

### 3.4. Boundary Limits Identification of Ammonia Removal and Operating Cost

The identification of bounds is essentially needed to determine the appropriate upper and lower boundary limits in the removal of the contaminant (including its cumulative uncertainty) and the total operating cost under the electrochemical oxidation of ammonia. The subsequent results in the process system are based on the simultaneous utilization of the generated BBD equation in the RSM and the cost equation. This would later be able to aid in the decision-making analysis in order to enable a quantifiable basis for its preference criterion selection. In the optimization analysis of this case study, non-linear model equations are employed that yield to numerous local optimum solutions. Thus, a global optimum solver using the Lingo software is vital in reaching the global optimum results based on its objective function subjected to various constraints [38]. 

A boundary limit analysis for the electrochemical oxidation of ammonia utilizes Equations (16)–(33). The model equations in Equations (16) and (17) are based on the validated models of Li et al. [10] that describe the production of ammonia and nitrates, respectively [10]. A cumulative uncertainty of response variables determined through its partial derivatives with respect to its each parametric factor has been added for ammonia in Equations (18)–(21) and for nitrates in Equations (22)–(25). Furthermore, the total operating cost in Equation (26), which is the sum of the material cost in Equation (27) and electric cost in Equation (28), plays an essential role in the boundary limit analysis. Energy consumption (directly related to the electric cost) is calculated in Equation (29). Finally, Equations (30)–(32) indicate the feasible regions for the variables of the NaCl dosage, current density, and electrolysis time, respectively.

(16) $$Y_{A}=175.1-28.59X_{1}-3.731X_{2}-1.331X_{3}+0.3019X_{1}X_{2}+0.1025X_{1}X_{3}+1.40\times 10^{-2}X_{2}X_{3}+1.088X_{1}^{2}\;+1.931\times 10^{-2}X_{2}^{2}+2.407\times 10^{-3}X_{3}^{2}$$

(17) $$Y_{N}=-27.39+0.7946X_{1}+1.212X_{2}+0.4180X_{3}+7.938\times 10^{-2}X_{1}X_{2}+3.056\times 10^{-2}X_{1}X_{3}-2.778\times 10^{-3}X_{2}X_{3}\;-0.2331X_{1}^{2}-1.721\times 10^{-2}X_{2}^{2}-2.498\times 10^{-3}X_{3}^{2}$$

(18) $$W_{Y_{A}}=\sqrt{{\left(\frac{\partial Y_{A}}{\partial X_{1}}W_{X_{1}}\right)}^{2}+{\left(\frac{\partial Y_{A}}{\partial X_{2}}W_{X_{2}}\right)}^{2}+{\left(\frac{\partial Y_{A}}{\partial X_{3}}W_{X_{3}}\right)}^{2}}$$

(19) $$\frac{\partial Y_{A}}{\partial X_{1}}=-28.59+0.3019X_{2}+0.1025X_{3}+2.175X_{1}$$

(20) $$\frac{\partial Y_{A}}{\partial X_{2}}=-3.731+0.3019X_{1}+1.40\times 10^{-2}X_{3}+3.863\times 10^{-2}X_{2}$$

(21) $$\frac{\partial Y_{A}}{\partial X_{3}}=-1.331+0.1025X_{1}+1.40\times 10^{-2}X_{2}+4.814\times 10^{-3}X_{3}$$

(22) $$W_{Y_{N}}=\sqrt{{\left(\frac{\partial Y_{N}}{\partial X_{1}}W_{X_{1}}\right)}^{2}+{\left(\frac{\partial Y_{N}}{\partial X_{2}}W_{X_{2}}\right)}^{2}+{\left(\frac{\partial Y_{N}}{\partial X_{3}}W_{X_{3}}\right)}^{2}}$$

(23) $$\frac{\partial Y_{N}}{\partial X_{1}}=0.7946+7.938\times 10^{-2}X_{2}-3.056\times 10^{-2}X_{3}-0.4663X_{1}$$

(24) $$\frac{\partial Y_{N}}{\partial X_{2}}=1.212+7.938\times 10^{-2}X_{1}-2.778\times 10^{-3}X_{3}-3.441\times 10^{-2}X_{2}$$

(25) $$\frac{\partial Y_{N}}{\partial X_{3}}=0.4180+3.056\times 10^{-2}X_{1}-2.778\times 10^{-3}X_{2}-4.996\times 10^{-3}X_{3}$$

(26) $$C_{T}=C_{M}+C_{E}$$

(27) $$C_{M}=2.28X_{1}$$

(28) $$C_{E}=0.18E_{C}$$

(29) $$E_{C}=\frac{10X_{2}X_{3}}{100-Y_{A}}$$

(30) $$1\leq X_{1}\leq 5$$

(31) $$10\leq X_{2}\leq 50$$

(32) $$30\leq X_{3}\leq 120$$

Global optimal solutions for the upper boundary limits of the electrochemical reaction system are determined in the subsequent maximization of the objective function related to ammonia removal in Equations (16) and (18) subjected to the constraints in Equations (17) and (19)–(32). This led to a complete removal of the contaminant and attained a total operating cost of USD 64.0 in the conditions of 5.0 NaCl dosage, 14.0 mA/cm^2^ current density, and 120.0 min electrolysis time. Conversely, the global optimum by the minimization of the cost parameter objective function in Equation (26) subjected to the constraints in Equations (16)–(25) and (27)–(32) draws out the lower boundary limits for the removal of ammonia and its total operating cost. Results indicate a total operating cost equivalent to USD 17.1 that reached only 40.6% ammonia removal. The conditions of the tested factors are as follows: 3.8 g NaCl dosage, 10.0 mA/cm^2^ current density, and 33.4 min electrolysis time.

It is observed that ammonia removal and the energy consumption increase with the total operating cost. This implies that a more intensive parametric condition is required in order to efficiently remove the ammonia contaminant in the system. This is also in agreement with the findings of Hansen et al. [35], wherein the energy needed rises along with the amount of contaminant removal in an electrochemical reaction. Furthermore, the increase in the total operating cost translates to the necessity of higher operating conditions. This directly contributes to additional costs with regards to its material usage and energy requirement that essentially improves the removal of ammonia.

### 3.5. Multi-Objective Decision Analysis: Fuzzy Optimization

**Figure 6:**
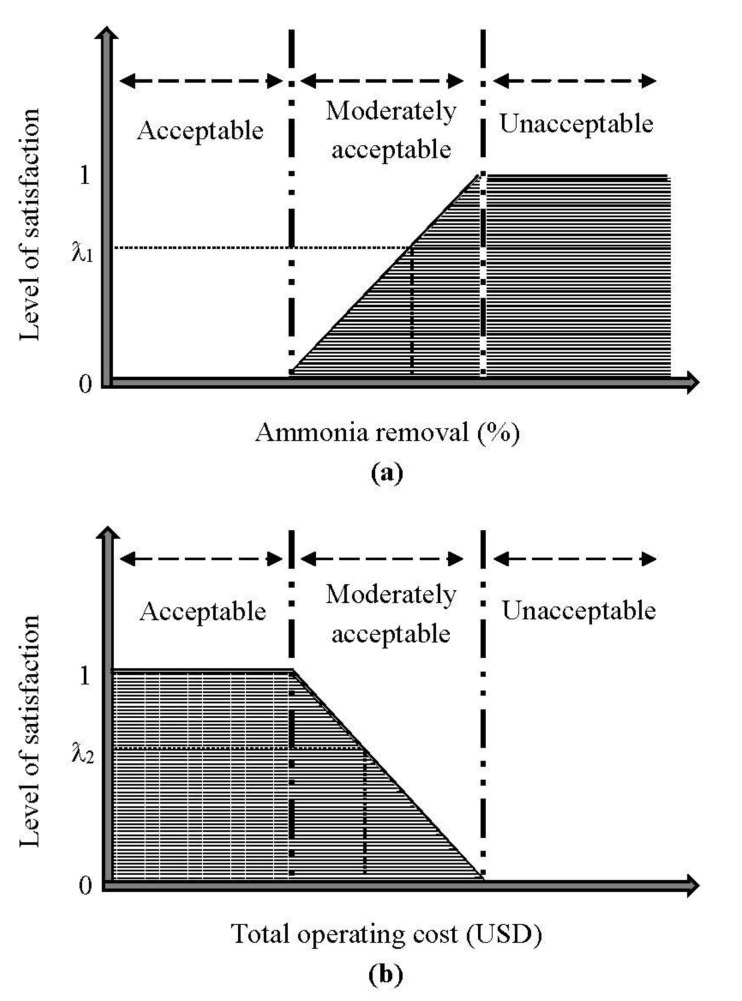
Linear membership function for the (**a**) ammonia removal and (**b**) total operating cost.

In this case study, a strategic decision-making analysis is performed by integrating the concept of fuzzy optimization for the electrochemical oxidation of ammonia. Specifically, a global optimization in maximizing the degree of satisfaction in Equation (33) as its objective function is subjected to the fuzzy constraints in Equations (34)–(36). In the fuzzy optimization process, the approximation and favorability of the non-linear equations used in this case study employ the frame of the linear membership function in order to bridge the gap between subjective and objective uncertainty in the decision-making process [39]. This brings about the illustrative explanation of the application of the linear membership function in the maximization of ammonia removal (Equation (34)) and the minimization of the total operating cost (Equation (35)) in Figure 6. The satisfaction levels for the results in ammonia removal and the total operating cost must comply with the overall degree of satisfaction in the selected criterion for the electrochemical process system. Furthermore, the calculated boundary limits (ammonia removal: 0% to 108.0%; total operating cost: USD 17.1 to 64.0) is the underlying basis for the multi-objective fuzzy optimization analysis in this case study. The unacceptable and acceptable ratings are quantified as “0” and “1”, respectively, and specifically equated in Equation (36).

(33) $$\lambda_{O}\leq \lambda_{1}\;\&\;\lambda_{2}$$

(34) $$\lambda_{1}=\frac{Y-40.65}{67.40}$$

(35) $$\lambda_{2}=\frac{64.02-C_{T}}{46.93}$$

(36) $$0\leq \lambda_{O}\leq 1$$

For this case study, the adherence on compliance with the stringent contaminant level standards is based on the USEPA. Thus, additional criterion for the multi-objective fuzzy optimization are added as follows: (1) Ammonia content ≤ 17 or 1.9 mg/L [5] and (2) nitrate content ≤ 10 mg/L [3]. This would aid in the selection of a proper basis for the fuzzy optimal solution. The fuzzy optimal solution is obtained in the simultaneous maximization of ammonia removal and minimization of the total operating cost.

Table 3 lists the simulated results of the fuzzy optimal solution for the electrochemical oxidation of ammonia. Upon the determination of the global maximization of the overall satisfaction $\left(\lambda_{O}\right)$, results showed a high satisfaction rating of 80.3% and 76.1% for adhering to the ammonia concentration standards of 10 and 1.9 mg/L, respectively. A slightly lower overall satisfaction rating is observed in compliance with a lower ammonia standard concentration due to the additional incurring costs that compromise the results. In setting the concentration limit of ammonia and nitrate to adhere at 10 mg/L, this ensued the individual satisfaction ratings of ammonia removal and total operating cost at 80.3% and 85.9%, respectively. In these criteria, the parametric conditions of 5.0 g NaCl, 10.0 mA/cm^2^ current density, and 64.7 electricity time yielded an ammonia removal and nitrate production with a certainty of 94.8% and 9.8 mg/L, respectively, at a total operating cost of USD 23.7. It is noted that the optimized results obtained an uncertainty range from 93.6% to 96.0% and from 9.6 to 10.0 mg/L for ammonia removal and nitrate production, respectively. On the other hand, setting the concentration of ammonia and nitrate to adhere at 1.9 and 10 mg/L, respectively, has led to the individual degrees of satisfaction of 86.7% (ammonia removal) and 76.1% (total operating cost). A higher satisfaction rating for the ammonia removal has been achieved as opposed to the previous criteria due to the attainment of higher ammonia removal with a certainty of 99.1% and an uncertainty range of 98.2% to 100.0%. Conversely, a lower satisfaction for the total operating cost as opposed to the previous criteria was attributed to a higher incurring cost in association with the added energy consumption requirement. 

### 3.6. Summary of Results

The individual parametric trend resulted in the highest ammonia removal at 5.0 g NaCl dosage, 50 mA/cm^2^ current density, and 120 min electrolysis time. On the other hand, the highest total operating cost was obtained at 1.0 g NaCl dosage, 50 mA/cm^2^ current density, and 30 min electrolysis time for the separately tested variables. This implies that the highest tested level for each parameter is the best for the electrochemical oxidation of ammonia, while its operating cost is dependent on the combination of the material usage and energy consumption. Based on the boundary limit identification, the upper and lower bounds for the ammonia removal were at 108.0% and 0%, respectively, while the upper and lower bounds for the total operating cost were at USD 64.0 and 17.1, respectively. After utilizing the results in the boundary limits, the fuzzy optimization resulted in the degree of satisfaction of 80.3% and 76.1% for adhering with the 10 and 1.9 mg/L ammonia level at its lowest cost, respectively. The fuzzy optimal solution for a 10 mg/L ammonia reached a removal rate of 94.8% ± 1.2% and USD 23.7 operating cost at the conditions of 5.0 g NaCl, 10.0 mA/cm^2^, and 64.7 min. For the 1.9 mg/L ammonia limit, 99.1% ± 0.9% ammonia removal and USD 28.3 total operating cost were achieved at 3.36 NaCl dosage, 10.0 mA/cm^2^, and 64.7 min.

In comparison with the optimal solutions based on the study of Li et al. [10], its total operating cost was computed to be approximately USD 78.8. This is 2.8 to 3.3 times higher than the results obtained in the current case study. Henceforth, this proves to show that the incorporation of the fuzzy optimization mathematical programming is essential towards its application in the RSM technique. This also supports the viability and practicality of fuzzy optimization in the decision-making process, upon determining the best ammonia removal at the lowest possible total operating cost in the process system of electrochemical reactions.

## 4. Conclusions

The case study of this research work incorporates the utilization of fuzzy optimization in order to appropriately determine the best compromise result in the electrochemical oxidation of ammonia, which satisfies various constraints in the process system. The decision-making analysis was based on the systematic approach of the simultaneous maximization of the removal of ammonia at the lowest possible cost in accordance of the parameters of the NaCl dosage, current density, and electrolysis time. This is in adherence with stringent environmental regulations set by USEPA for ammonia and nitrate concentrations. The boundary limits of ammonia removal with the inclusion of its cumulative uncertainty criteria (40.6% ± 2.2% to a complete removal) and total operating cost (USD 17.1 to 39.4) was realized in the boundary limit analysis. The application of fuzzy optimization resulted in an overall satisfaction rating of greater than 76% that implies a proper allocation towards a good compromise result between ammonia removal and cost. A decrease in the satisfaction rating showed a higher cost in the consumed energy needed to remove ammonia effectively. In comparison with using RSM alone in the electrochemical oxidation of ammonia, the integration of the fuzzy mathematical programming optimization showed a lower costing to about 178.4% to 232.5%. Thus, this case study has successfully drawn-out promising results that can be further extended or applied in future environmental applications in the process system of the electrochemical reaction.

The limitations of this research study are in the utilized process parameters. Specifically, the model equations used are limited to only one electrolyte (i.e., NaCl) at levels from 1 to 5 g. Therefore, a comparative assessment of various electrolytes at a wider range (e.g., 0.1 to 10 g) is recommended to further improve the analysis of the electrolyte influence on the electrochemical oxidation of ammonia. Other important parameters such as solution pH, ammonia concentration, and temperature can also be explored in future fuzzy optimization studies in order to assess their effects on electrolysis efficiency, energy consumption, and operating cost. Lastly, a comparative study of the application of Pareto solutions utilizing a weighting method and the ε-constraint method can be used for an intricate analysis in the future research outlook of the modeling technique in the electrochemical oxidation of ammonia.

## Figures and Tables

**Table 1:** (**a**) Process and (**b**) cost factors of the electrochemical oxidation of ammonia.

(a) Process factors and Scale			
Variables	Scale of the Experiment		
	Unit	Range	
Sodium chloride (*X*_1_)	g	1–5	
Current density (*X*_2_)	mA/cm^2^	10–50	
Electrolysis time (*X*_3_)	min	30–120	
**(b) Cost factors**	**Unit**	**Price**	**Source**
Material cost (sodium chloride)	USD/g	2.28	Sigma-Aldrich
Energy consumption cost	USD/kWh	0.18	Meralco (December 2020)

**Table 2:** Ammonia removal by the electro-oxidation process: Box-Behnken design matrix.

Run	Sodium Chloride (*X*_1_: g)	Current Density (*X*_2_: mA/cm^2^)	Electrolysis Time (*X*_3_: min)	Unit: %	
				Ammonia Removal	Cumulative Uncertainty *
1	1	10	75	52.86	±2.36
2	1	30	30	59.99	±2.15
3	1	30	120	100.27	±0.76
4	1	50	75	101.69	±0.55
5	3	10	30	51.55	±2.40
6	3	10	120	98.58	±1.01
7	3	30	75	100.00	±0.80
8	3	30	75	100.00	±0.80
9	3	30	75	100.00	±0.80
10	3	30	75	100.00	±0.80
11	3	30	75	100.00	±0.80
12	3	50	30	101.42	±0.59
13	3	50	120	98.06	±0.80
14	5	50	75	98.84	±0.76
15	5	30	30	99.74	±0.84
16	5	30	120	103.12	±0.55
17	5	10	75	98.31	±1.05

* Cumulative uncertainty of the results (computed).

**Table 3:** Fuzzy optimal results in the electro-oxidation process based on maximum contaminant levels (MCL) in United States Environmental Protection Agency.

Parameters	Unit	MCL: 10 mg/L NH_3_-N	MCL: 1.9 mg/L NH_3_-N
$\lambda_{O}$	%	80.3	76.1
$\lambda_{Ammonia}$	%	80.3	86.7
$\lambda_{Cost}$	%	85.9	76.1
$\mathrm{Ammonia}\;\mathrm{removal}\;\left(Y_{A}\right)$	%	94.8	99.1
$\mathrm{Cumulative}\;\mathrm{uncertainty}\;\left(W_{Y_{A}}\right)$	%	1.2	0.9
Energy consumption	kWh/kg-NH_3_	68.3	114.7
Material cost	USD	11.4	7.7
Electric cost	USD	12.3	20.6
Total operating cost	USD	23.7	28.3
$\mathrm{Nitrate}\;\mathrm{production}\;\left(Y_{N}\right)$	mg/L	9.8	9.5
$\mathrm{Cumulative}\;\mathrm{uncertainty}\;\left(W_{Y_{N}}\right)$	mg/L	0.2	0.5
Sodium chloride	g	5.0	3.36
Current density	mA/cm^2^	10.0	10.0
Electrolysis time	min	64.7	113.7
